# Supplementary material for: Efficient high-precision homology-directed repair-dependent genome editing by HDRobust
Source: Nat Methods. 2023 Jul 20;20(9):1388–99. doi: 10.1038/s41592-023-01949-1 (PMC10482697; doi:10.1038/s41592-023-01949-1)
Supplement: Supplementary file 1 — Supplementary discussion and Fig. 1. [file 41592_2023_1949_MOESM1_ESM.pdf]

nature methods

Article https://doi.org/10.1038/s41592-023-01949-1

Efficient high-precision homology-directed repair-dependent genome editing by HDRobust

In the format provided by the authors and unedited

Supplementary Information

Supplementary Discussion

Genome stability when NHEJ and MMEJ are inhibited by mutations. We have previously shown that the sole inhibition of NHEJ by DNA-PKcs K3753R does not increase aneuploidy, chromosomal rearrangements, or mutations per passage [20] and mice lacking *polq* show a very mild enhanced chromosome instability phenotype, develop normally and are fertile [88]. To investigate the genome stability of H9 hESCS carrying the DNA-PKcs K3753R and PolΘ V896* mutations, we cultured them in media containing a sub-lethal amount of bleomycin to mimic spontaneous DSBs that occur during long-term cell culture as well as DSBs occurring after repeated CRISPR cleavage. After five months, spectral karyotyping (SKY) and Giemsa banding (GTG) were performed. Both wild type H9 hESCs and the double repair mutant hESCS contained around 2% abnormal metaphases indicating that the genome stability is not compromised in double repair mutant cells relative to wild type cells (Supp. Fig. 1).

Supplementary Figure

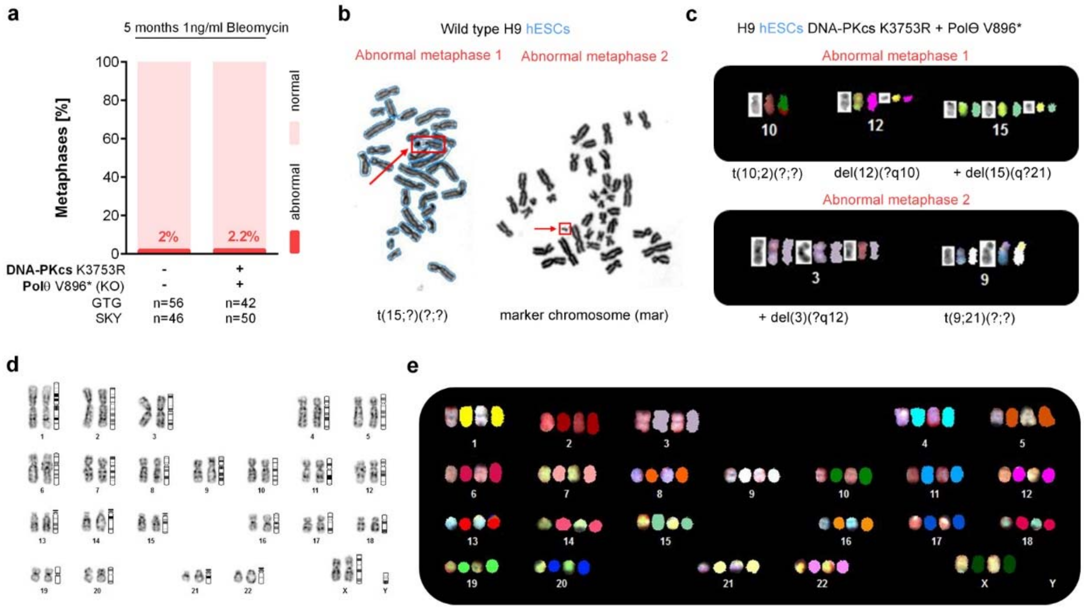

Supplementary Fig. 1: Karyotype analysis after long-term culture with bleomycin. (**a**) Percentage of normal and abnormal metaphases of H9 hESCs that carry no repair gene mutation (wild type) or DNA-PKcs K3753R and PolΘ V896* (double mutant) after five months of cell culture with 1ng/ml bleomycin, which is the highest amount tested that allowed propagation of both cell lines. The number of metaphases analyzed by spectral karyotyping (SKY) and Giemsa banding (GTG) are stated. Images of altered chromosomes in both observed abnormal metaphases in wild type (**b**) and double mutant (**c**). An example of the majority of normal metaphases observed when characterized by GTG (**d**) and SKY (**e**).
