# Supplementary material for: Efficient high-precision homology-directed repair-dependent genome editing by HDRobust
Source: Nat Methods. 2023 Jul 20;20(9):1388–99. doi: 10.1038/s41592-023-01949-1 (PMC10482697; doi:10.1038/s41592-023-01949-1)
Supplement: Supplementary file 2 — Reporting Summary [file 41592_2023_1949_MOESM2_ESM.pdf]

nature portfolio

Corresponding author(s): Stephan Riesenberg

Last updated by author(s): Apr 28, 2023

# Reporting Summary

Nature Portfolio wishes to improve the reproducibility of the work that we publish. This form provides structure for consistency and transparency in reporting. For further information on Nature Portfolio policies, see our Editorial Policies and the Editorial Policy Checklist.

## Statistics

For all statistical analyses, confirm that the following items are present in the figure legend, table legend, main text, or Methods section.

| n/a | Confirmed | |
|---|---|---|
| ☐ | ☒ | The exact sample size (*n*) for each experimental group/condition, given as a discrete number and unit of measurement |
| ☐ | ☒ | A statement on whether measurements were taken from distinct samples or whether the same sample was measured repeatedly |
| ☒ | ☐ | The statistical test(s) used AND whether they are one- or two-sided<br>*Only common tests should be described solely by name; describe more complex techniques in the Methods section.* |
| ☒ | ☐ | A description of all covariates tested |
| ☒ | ☐ | A description of any assumptions or corrections, such as tests of normality and adjustment for multiple comparisons |
| ☐ | ☒ | A full description of the statistical parameters including central tendency (e.g. means) or other basic estimates (e.g. regression coefficient) AND variation (e.g. standard deviation) or associated estimates of uncertainty (e.g. confidence intervals) |
| ☒ | ☐ | For null hypothesis testing, the test statistic (e.g. $F$, $t$, $r$) with confidence intervals, effect sizes, degrees of freedom and $P$ value noted<br>*Give P values as exact values whenever suitable.* |
| ☒ | ☐ | For Bayesian analysis, information on the choice of priors and Markov chain Monte Carlo settings |
| ☒ | ☐ | For hierarchical and complex designs, identification of the appropriate level for tests and full reporting of outcomes |
| ☒ | ☐ | Estimates of effect sizes (e.g. Cohen's $d$, Pearson's $r$), indicating how they were calculated |

*Our web collection on statistics for biologists contains articles on many of the points above.*

## Software and code

Policy information about availability of computer code

| | |
|---|---|
| Data collection | No software was used. |
| Data analysis | CRISPResso (v1), SAMtools (v1.12), GraphPad Prism (v6), Adobe Photoshop (CS5), ImageJ (v1.53) |

For manuscripts utilizing custom algorithms or software that are central to the research but not yet described in published literature, software must be made available to editors and reviewers. We strongly encourage code deposition in a community repository (e.g. GitHub). See the Nature Portfolio guidelines for submitting code & software for further information.

## Data

Policy information about availability of data

All manuscripts must include a data availability statement. This statement should provide the following information, where applicable:

- Accession codes, unique identifiers, or web links for publicly available datasets
- A description of any restrictions on data availability
- For clinical datasets or third party data, please ensure that the statement adheres to our policy

The sequencing data generated in this study have been deposited in the Dryad database under accession code dryad.fj6q5740f. Data are also available on request from authors.

## Human research participants

Policy information about studies involving human research participants and Sex and Gender in Research.

| | |
|---|---|
| Reporting on sex and gender | N/A |
| Population characteristics | N/A |
| Recruitment | N/A |
| Ethics oversight | N/A |

Note that full information on the approval of the study protocol must also be provided in the manuscript.

# Field-specific reporting

Please select the one below that is the best fit for your research. If you are not sure, read the appropriate sections before making your selection.

☒ Life sciences ☐ Behavioural & social sciences ☐ Ecological, evolutionary & environmental sciences

For a reference copy of the document with all sections, see nature.com/documents/nr-reporting-summary-flat.pdf

# Life sciences study design

All studies must disclose on these points even when the disclosure is negative.

| | |
|---|---|
| Sample size | No sample size calculation was performed. At least two independent biological replicates for each condition were obtained. This was sufficient as the editing efficiency difference between replicates of a respective group was very small compared to the strong difference observed between the compared groups. |
| Data exclusions | No data were exluded from analysis. |
| Replication | All attempts of replication were successful. At least two independent biological replicates for each condition were obtained. |
| Randomization | Mammalian cells used in this study were cells grown under identical conditions and experiments were done in parallel. Thus, no randomization was used. |
| Blinding | Mammalian cells used in this study were cells grown under identical conditions and experiments were done in parallel. Thus, blinding was not used for data collection. Analysis was performed based on numerical sample names, without the identity of the samples known during the analysis. |

# Reporting for specific materials, systems and methods

We require information from authors about some types of materials, experimental systems and methods used in many studies. Here, indicate whether each material, system or method listed is relevant to your study. If you are not sure if a list item applies to your research, read the appropriate section before selecting a response.

### Materials & experimental systems

| n/a | Involved in the study | |
|---|---|---|
| ☒ | ☐ | Antibodies |
| ☐ | ☒ | Eukaryotic cell lines |
| ☒ | ☐ | Palaeontology and archaeology |
| ☒ | ☐ | Animals and other organisms |
| ☒ | ☐ | Clinical data |
| ☒ | ☐ | Dual use research of concern |

### Methods

| n/a | Involved in the study | |
|---|---|---|
| ☒ | ☐ | ChIP-seq |
| ☒ | ☐ | Flow cytometry |
| ☒ | ☐ | MRI-based neuroimaging |

## Eukaryotic cell lines

Policy information about cell lines and Sex and Gender in Research

| | |
|---|---|
| Cell line source(s) | H9 hESCs (WiCell Research Institute, WA09), 409-B2 hiPSCs (Riken BioResource Center, HPS0076), K562 cells (ECACC, |

| | |
|---|---|
| Cell line source(s) | 89121407), CD4+ T cells (HemaCare, PB04C-1), Lymphoblastoid cells (Coriell Institute, GM14890, HG02367, GM16265, GM08369) |
| Authentication | All cell lines were authenticated by the supplier via certificate of analysis and additionally in-house by checking morphology. |
| Mycoplasma contamination | All cell lines were tested negative for mycoplasma contamination before and after the experiments. |
| Commonly misidentified lines (See ICLAC register) | No commonly misidentified cell lines were used in the study. |
